# Atypical Thyroid Function Tests and Thyroid Hormone Resistance

**DOI:** 10.7759/cureus.16328

**Published:** 2021-07-12

**Authors:** Vishwanath Pattan, Ishita Mehra, Syed Anjum Khan, Rahul Kashyap

**Affiliations:** 1 Endocrinology, Wyoming Medical Center, Casper, USA; 2 Internal Medicine, North Alabama Medical Center, Florence, USA; 3 Critical Care Medicine, Mayo Clinic Mankato, Mankato, USA; 4 Critical Care Medicine, Mayo Clinic, Rochester, USA

**Keywords:** thyroid hormone resistance, thyroid hormone receptor beta, atypical thyroid function tests, autosomal dominant, artifactual lab interference

## Abstract

Clinical interpretation of thyroid labs is usually straightforward. However, there are rare instances when the atypical profile of thyroid labs warrants systematic investigation to determine the underlying cause. We report the case of a 90-year-old Caucasian male with a chronic history of atrial fibrillation with chronic pacemaker dependence who presented with significantly elevated free thyroxine level (>7.77 ng/dL) but normal thyroid-stimulating hormone level (2.15 µIU/mL). After ruling out pituitary tumors and artifactual errors due to lab interference, the diagnosis of thyroid hormone resistance was made.

## Introduction

Thyroid labs are one of the most commonly ordered lab tests by primary care physicians. On most occasions, interpretation of thyroid labs is straightforward. However, on rare occasions, primary physicians encounter thyroid lab panels that do not make sense. Normally thyrotropin-releasing hormone (TRH) from the hypothalamus stimulates the production of thyroid-stimulating home (TSH) which, in turn, acts on the thyroid gland to regulate the synthesis and release of tetraiodothyronine (T4) and triiodothyronine (T3). Three carrier proteins, namely, albumin, thyroxine-binding prealbumin, and thyroxine-binding globulin (TBG) bind most of the circulating thyroid hormones in the blood and only a small fraction are available as free hormones (about 0.3% T3 and 0.02% T4) [1]. The secreted T4 and T3 (especially T3) provide inhibitory feedback to both hypothalamic TRH and pituitary TSH secretion, thus completing the loop. Therefore, the levels of TSH decrease below the normal range when the levels of T4 and T3 are significantly elevated. However, there are rare exceptions to this rule. In this case report, we discuss a very rare and challenging diagnostic dilemma posed by a 90-year-old Caucasian male who had significantly elevated T4 levels but had normal TSH levels.

## Case presentation

A 90-year-old Caucasian male with a history of atrial fibrillation, hypertension, and primary hypogonadism was referred to endocrinology for evaluation of abnormal thyroid labs and suspected hyperthyroidism.

The patient had a thyroid lab panel ordered routinely which was found to be significantly abnormal. Labs showed TSH of 2.15 µIU/mL (0.46-4.68 µIU/mL), free T4 of >7.77 ng/dL, total T4 of 19.5 µg/dL (reference range: 4.5-12 µg/dL), and free T3 of 5 pg/mL (reference range: 2.0-4.4 pg/mL). He was not taking any biotin supplement and did not receive any iodinated contrast before the labs. The patient had a history of atrial fibrillation and had undergone radiofrequency ablation in 1991. He had been on apixaban and testosterone replacement but not on amiodarone, vitamins, or biotin supplements before the lab draw. The patient was asymptomatic. He had been completely dependent on a pacemaker for his heart rate. His heart rate varied from 70-130 beats per minute, and it was programmed for dynamic changes during physical activity and exercise. The patient denied tremors, palpitations, and heat intolerance.

The patient had a history of hypogonadism. He got hot sweats if he missed his testosterone replacement dose. None of his family members had abnormal thyroid blood tests, including his two sons. Physical examination showed a heart rate of 96 beats per minute, blood pressure 136/86 mmHg, respiratory rate 16 breaths per minute, oxygen saturation 97% on room air, height 180.3 cm, weight 91.5 kg, and body mass index of 28. The thyroid gland was not palpable. The rest of the physical examination was unremarkable except for bilateral atrophic testicles.

The patient had a mumps infection in the mid-20s during which one of the testicles was inflamed. Subsequently, he had a minor hernia repair, following which the other testicle also atrophied. Thyroid-stimulating immunoglobulin and thyrotropin receptor antibody levels were undetectable. The patient could not get an MRI because of the pacemaker. CT of the pituitary gland was normal. A summary of pertinent lab workup is provided in Table 1. Workup for anterior pituitary hormone deficiency was negative. Further workup that was recommended included a T4 binding assay (which measures all three binding proteins and the percentage binding of T4 to each) to see if there was an abnormal binding protein. However, the patient declined further workup.

**Table 1:** Summary of lab workup. RTH: resistance to thyroid hormone; TSH: thyroid-stimulating hormone; T4: tetraiodothyronine; T3: triiodothyronine

Lab values (reference range) and time	TSH (0.46-4.68 µIU/mL)	Free T4 (0.76-1.46 ng/dL)	Free T3 (2.0-4.4 pg/mL)	Total T4 (4.5-12 µg/dL)	Human anti-mouse antibody (0-74 ng/mL)	RTH gene sequencing	Alpha subunit (reference range: <2.13 ng/mL)	Prolactin (4.0-15.2 ng/mL)
Initial labs in 2019	2.15	>7.77	-	19.5	-	-	-	-
4 months later	2.11	>6.5	16.05	-	-	-	-	-
4.5 months later	1.8	>7.77	-	-	<56	-	-	-
5 months later	-	-	-	-	-	-	0.86	9.1
5.5 months later	1.82	>7.77	5.4	-	-	-	-	-
11 months later	2.34	>7.77	5.9	-	-	-	-	-
12 months later	-	-	-	-	-	Negative	-	-

## Discussion

Our patient had an elevated free T4 level in the presence of normal TSH, which, in the absence of a pituitary tumor or evidence of artifactual errors in lab assay, is suspicious for thyroid hormone resistance. However, our patient did not have any family history suggestive of thyroid hormone resistance. Many medications can also affect thyroid labs [2-4]. He was not taking any medications, biotin, or had any iodinated contrast that could have affected thyroid labs. Although T4 binding assay was not performed to determine abnormal binding proteins, these are unlikely to affect the free T4 levels. Thus, in the absence of other causes of atypical thyroid lab profile of elevated free T4 and normal TSH, a diagnosis of thyroid hormone resistance was made. Thyroid hormone resistance is usually an autosomal dominant inherited syndrome. The most common defect in thyroid hormone action is a mutation of the *thyroid hormone receptor beta* (*THRβ*) gene. Although the majority of *TRβ*-mediated thyroid hormone resistance is inherited as autosomal dominant, up to 20% can occur sporadically due to de novo mutations. In addition, negative *THRβ* gene sequencing does not exclude *THRβ* defects.

The thyroid hormones are responsible for the regulation of various physiological functions by binding to four identified thyroid hormone receptors isoforms, namely, thyroid hormone receptor α1 (THRα1), thyroid hormone receptor α2 (THRα2), thyroid hormone receptor β1 (THRβ1), and thyroid hormone receptor β2 (THRβ2). THRβ1 is expressed in the liver, kidney, and thyroid and THRβ2 is expressed in the pituitary, hypothalamus, and ear [5]. THRβ2 has been thought to affect the negative feedback mechanism to the hypothalamus-pituitary-thyroid axis [6]. Resistance to thyroid hormone beta (RTHβ) was first described in 1967 [7], and while familial autosomal dominant and autosomal recessive *THRβ *mutations have been described in the majority of the cases, up to 20% of cases can be due to de novo mutations [5,8]. These mutations should be suspected in the context of elevated free T3 and T4, normal or elevated TSH, and normal thyrotropin receptor antibody (TRAb) levels. These derangements in the thyroid function lead to a constellation of clinical abnormalities, including, but not limited to, goiter, tachycardia, short stature, hyperactivity, developmental delay, and hearing impairment, and this is further dependent on the affected thyroid receptor isoform [9-11].

The incidence of RTH has not been clearly defined; however, a study conducted in Oregon reported an incidence of 1 in 40,000 live births and approximately 1,000 cases have been reported in the literature between 1967 and 2006 [11]. Where genetic testing is regarded as the gold standard required for the diagnosis of RTHβ, case reports have brought forward evidence of mosaicism causing varied phenotypical manifestations of RTHβ within the family without any evident mutations in the *THRβ* gene [12]. Approximately 10% of all RTH is secondary to unknown causes or factors such as the decreased number of receptors and is not attributed to gene mutations [8]. RTHβ can be further classified into general, peripheral, and pituitary types, where peripheral RTHβ can have symptoms suggestive of hypothyroidism and pituitary RTHβ may appear to be causing hyperthyroidism due to inappropriate secretion of TSH [13]. The treatment of RTHβ is challenging and often symptom-driven. For example, tachycardia can be treated with selective beta-blockers; high-dose levothyroxine can be considered for symptomatic hypothyroidism. Thyroid hormone reduction by itself is not recommended because it can increase TSH and lead to goiter. At present, triiodothyroacetic acid is considered to be the most promising treatment as it lowers both thyroid hormone and TSH levels [13]. Our patient was asymptomatic and did not need active management for thyroid hormone resistance.

## Conclusions

In the setting of normal TSH and elevated free T4, after ruling out the pituitary tumor and artifactual lab interference, a high index of clinical suspicion is needed to diagnose thyroid hormone resistance, in the absence of a family history of thyroid hormone resistance and negative *THRβ* gene mutation.
